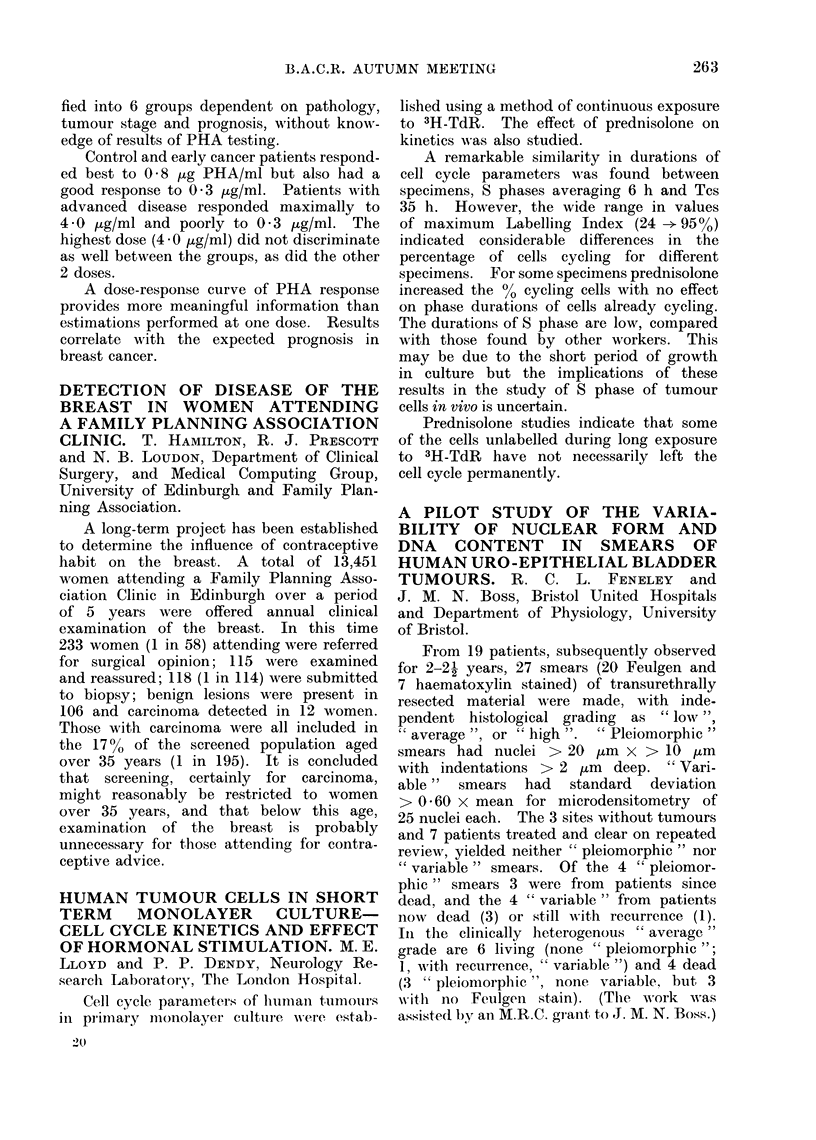# Proceedings: A pilot study of the variability of nuclear form and DNA content in smears of human uro-epithelial bladder tumours.

**DOI:** 10.1038/bjc.1975.48

**Published:** 1975-02

**Authors:** R. C. Feneley, J. M. Boss


**A PILOT STUDY OF THE VARIABILITY OF NUCLEAR FORM AND DNA CONTENT IN SMEARS OF HUMAN URO-EPITHELIAL BLADDER TUMOURS.** R. C. L. Feneley and J. M. N. Boss, Bristol United Hospitals and Department of Physiology, University of Bristol.

From 19 patients, subsequently observed for 2–2½ years, 27 smears (20 Feulgen and 7 haematoxylin stained) of transurethrally resected material were made, with independent histological grading as " low ", " average ", or " high ". " Pleiomorphic " smears had nuclei $> 20$ μm $\times > 10$ μm with indentations $> 2$ μm deep. " Variable " smears had standard deviation $> 0{\cdot}60 \times$ mean for microdensitometry of 25 nuclei each. The 3 sites without tumours and 7 patients treated and clear on repeated review, yielded neither " pleiomorphic " nor " variable " smears. Of the 4 " pleiomorphic " smears 3 were from patients since dead, and the 4 " variable " from patients now dead (3) or still with recurrence (1). In the clinically heterogenous " average " grade are 6 living (none " pleiomorphic "; 1, with recurrence, " variable ") and 4 dead (3 " pleiomorphic ", none variable, but 3 with no Feulgen stain). (The work was assisted by an M.R.C. grant to J. M. N. Boss.)